# COVID-19 Pandemic in Dialysis Patients: The Swiss Experience

**DOI:** 10.3389/fpubh.2022.795701

**Published:** 2022-05-17

**Authors:** Rebecca Guidotti, Menno Pruijm, Patrice M. Ambühl

**Affiliations:** ^1^Institute of Nephrology, Stadtspital Zürich, Zurich, Switzerland; ^2^Service of Nephrology and Hypertension, University Hospital of Lausanne and University of Lausanne, Lausanne, Switzerland

**Keywords:** COVID-19, SARS-CoV-2, pandemic, dialysis, incidence, regional differences, mortality

## Abstract

**Background:**

Chronic dialysis patients are classified as patients with increased risk for COVID-19. Knowledge about the incidence and survival of chronic dialysis patients infected with SARS-CoV-2 in Switzerland - a high-income country with high density of relatively small dialysis centers - is scarce. We present the findings regarding incidence, survival and regional differences, compared to those of the general population in Switzerland.

**Methods:**

Information on chronic dialysis patients who tested positive for SARS-CoV-2 between February 24, 2020 and February 28, 2022 were reported to the Swiss dialysis registry by all 94 Swiss dialysis centers. Hereafter, these results were linked with clinical characteristics from the Swiss dialysis registry.

**Results:**

Throughout the study period 1,120 out of ~4,700 dialysis patients tested positive for SARS-CoV-2 in Switzerland: 96 cases occurred in the first wave, 472 in the second wave and 5 in between. During the first wave, Italian-speaking Ticino was most severely affected, with a 7-fold higher incidence of dialysis patients compared to the general Swiss population. In the second wave, the majority of cases were found in the French-speaking part of Switzerland, with a 2.5 times higher incidence vs. non-dialysis patients. A total of 123 deaths were recorded in the first two waves, of which COVID-19 was the main cause of death in 100 patients. This corresponds to a highly increased overall mortality rate of 17.5% compared to 1.7% in the general population. Age was identified as the only risk factor for mortality in dialysis patients. During the third, fourth and fifth wave, 61, 43 and 443 cases, respectively, were recorded, resulting in 6 (mortality rate 9.8%), 1 (mortality rate 2.3%) and 13 deaths (mortality rate 2.9%).

**Conclusion:**

Chronic dialysis patients in Switzerland were more likely to be infected by SARS-CoV-2 during the first and second wave than the rest of the population, but an inverse trend was observed during the third, fourth and fifth wave, probably thanks to vaccination. In addition, mortality is significantly increased compared to non-dialysis patients. In Swiss dialysis patients, age is the strongest risk factor for death.

## Introduction

The first Severe Acute Respiratory Syndrome Coronavirus 2 (SARS-CoV-2) infection in Switzerland (CH) was confirmed on February 24, 2020, in the canton of Ticino, which borders northern Italy. Hereafter, the number of infections in the Swiss general population increased rapidly, and on March 16, due to the continuing increase in the number of infections, the Federal Council declared the situation as “extraordinary” (highest risk level) in accordance with the Epidemics Act (1), and ordered a complete lockdown.

**Figure 1:**
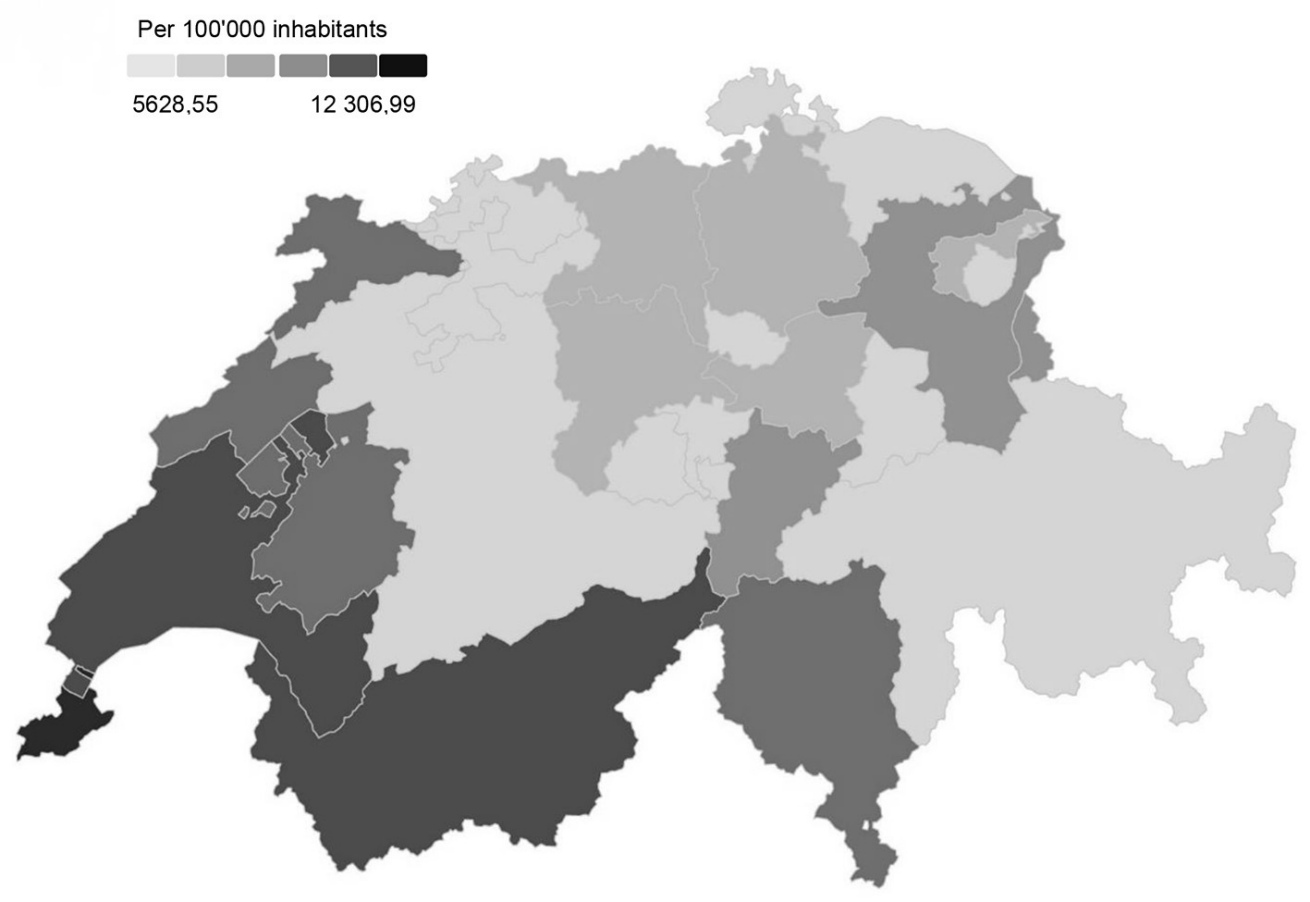
Overall geographical spread of SARS-CoV-2 cases / 100'000 inhabitants in the general population in Switzerland between 24.02.2020 and 19.05.2021 (5).

From February 24 until May 31, 2020 (end of first wave), Switzerland and the Principality of Liechtenstein (hereinafter collectively reported as “Switzerland”) counted a total of 31'029 confirmed SARS-CoV-2 infections, corresponding to an incidence rate of 3.7 cases per 100,000 person-days. In the Italian-speaking part of Switzerland (Ticino), the incidence rate was highest with 9.5 cases per 100,000 person-days, followed by the French-speaking part in western Switzerland with 6.8 and the German-speaking part with 2.2 cases per 100,000 person-days (2, 3). The numbers remained low during the summer months (June, July and August), only to rise sharply again at the end of September (4), marking the start of the second wave. Between October 1, 2020, and January 31, 2021 (second wave), 471,343 people in Switzerland tested positive for the coronavirus, which corresponds to an incidence rate of 44.3 cases per 100,000 person-days (2, 3). The overall geographical spread (including the first and second wave) of the SARS-CoV-2 cases in Switzerland is shown in Figure 1.

Since, like many other European countries, Switzerland has experienced a third, fourth and fifth wave, and the end of the epidemic is not in sight. Whereas incidence rates of the general population have been well documented and reported, data from the Swiss dialysis population are largely lacking, except for the canton of Geneva (6).

Patients on maintenance dialysis are at increased risk for severe coronavirus disease 2019 (COVID-19) and its complications (7, 8), due to the high prevalence of cardiovascular disease, diabetes, arterial hypertension, and age (9). Additionally, they are in close contact with health care providers. Moreover, hemodialysis patients are required to travel several times per week to and from the dialysis unit. In Switzerland, unlike many other countries, transport costs are only partially covered by health insurances. Therefore, hemodialysis patients often use public transportation, thus further exposing themselves to the risk of community-transmitted infection (10).

The aims of this study were to determine the incidence and course of SARS-CoV-2 infections in the Swiss dialysis population during the five waves, to identify and understand regional differences, and to compare the results to other European countries. The Swiss renal registry and quality assessment program (SRRQAP), established in 2006 by the Swiss Society of Nephrology, conducted this study.

## Methods

This prospective study was approved by the local ethics committee of the canton of Zurich, Switzerland (KEK-ZH), and performed in agreement with the declaration of Helsinki. This study is categorized as *further use of health-related personal data for research* according to the Swiss human research act.

### Cohort Population

All patients on chronic dialysis, hemodialysis (HD) and peritoneal dialysis (PD), with confirmed SARS-CoV-2 infection were included in this analysis. Since hemodialysis with over 90% of patients constitutes the predominant treatment modality in Switzerland Guidotti and Ambüh (Unpublished data), HD and PD patients were analyzed collectively.

### Instruments and Key Measures

At the end of March 2020, the SRRQAP distributed for the first time a questionnaire to all dialysis centers in Switzerland, asking the responsible nephrologist of each center to collect the number of confirmed cases with Covid-19 infection and number of subsequent deaths. The questionnaire captured (1) year of birth, (2) gender, (3) date of positive test, (4) death, (5) death of date and (6) cause of death. Furthermore, the registry identification number had to be provided, in order to link the test results with registry data and for quality control. Since, the survey was performed monthly from March 2020 onwards until the 28^th^ February 2022, with a follow-up of deaths in March 2022.

### Laboratory Testing

Positive cases were based on a reverse transcriptase-polymerase chain reaction (RT-PCR) test on samples obtained from the upper respiratory tract by nasopharyngeal or oropharyngeal swab. Since nephrologists function as the general practitioner for the dialysis patients in the Swiss health care system, most patients were referred for PCR testing to them. Positive tests results were therefore not based on reporting by the patients, but on tests performed or requested by nephrologists in charge.

### Data Acquisition and Statistical Analysis

For data base handling and analysis, we used Microsoft Excel® (Microsoft, USA) and IBM SPSS statistics application version 25 (IBM Corporation, USA). Baseline characteristics were expressed as mean±SD, median (interquartile range) or percentage, as appropriate. Comparisons between two groups were performed by Student's *t*-test for discrete variables or chi-squared tests for categorical variables. A *p*-value below 0.05 was considered statistically significant. To determine the risk factor for death in dialysis patients, we used a logistic regression model. Various variables (age, sex, ethnicity, BMI, region, diabetes, COPD, home dialysis and number of dialysis sessions per week) were inserted into the model and the final model with the best Akaike information criterion (AIC) was determined using backward elimination. Then a Cox proportional hazard model was used including the determined variables by the logistic regression, in order to investigate their impact on survival.

Survival analysis was carried out by Kaplan-Meier survival curve andtime in survival analysis was calculated from time of positive testing until death.

### Wave Classification

As there is no exact and uniform definition to classify a wave, we used tables from the Federal Office of Public Health to this purpose. The start of a wave begins with an exponential rise in positive cases and ends when they drop sharply.

## Results

### Centers

In Switzerland, over 4,700 patients underwent dialysis treatment in 2019. Most dialysis centers are located in the German speaking part of Switzerland (*N* = 63), whereas 24 are located in the French and seven in the Italian speaking part. All 94 centers in Switzerland contribute data to the registry. On March 27, 2020, all dialysis centers were invited to complete the questionnaire regarding incidence and outcome of COVID-19. None refused.

### First Wave (24.02.2020–31.05.2020)

#### Number of Patients and Distribution

**Figure 2:**
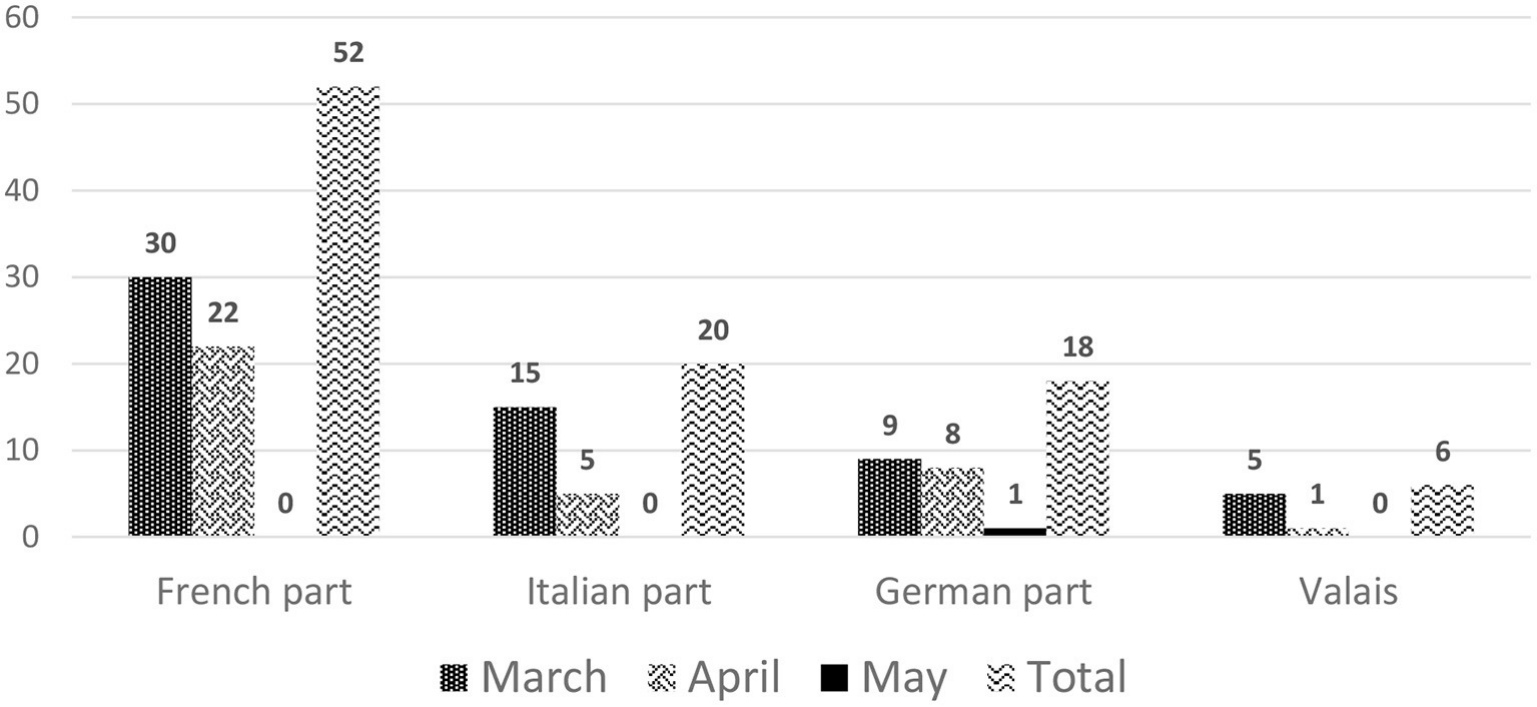
Number of dialysis patients who tested positive for SARS-CoV-2 stratified by month and region in the first wave.

**Figure 3:**
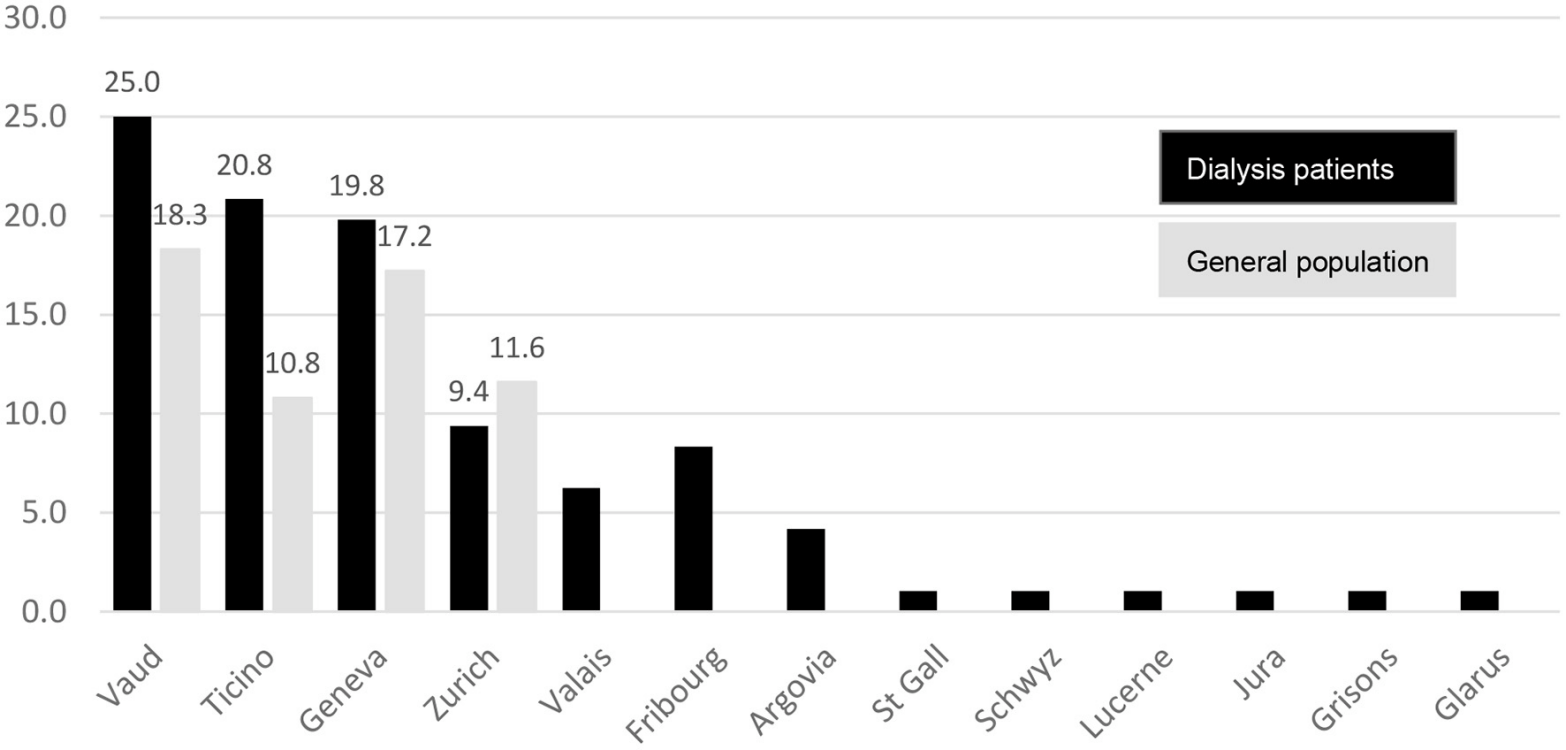
Percentage of positive SRARS-CoV-2 patients in each canton during the first wave in dialysis patients (black bars) and the general population (gray bars).

On March 5, 2020, the first SARS-CoV-2 infection in a dialysis patient occurred in Ticino. The number of infected dialysis patients increased rapidly over the months of March and April 2020, when 59 and 36 cases, respectively, were reported, as shown in Figure 2. Afterwards, the numbers decreased continuously, resulting in a total of 96 positive tested dialysis patients in the first wave. Thirty-four dialysis centers in 13 out of 26 cantons in Switzerland were affected with positive cases, mostly situated in the cantons of Vaud (25%), Ticino (20.8%) and Geneva (19.8%), which together made up for two-thirds of the SARS-CoV-2 infected dialysis patients in Switzerland during the first wave, as shown in Figure 3.

The Italian-speaking part accounted for the highest incidence rate with 66.9 cases per 100,000 person-days, closely followed by the French-speaking regions with an incidence rate of 61.1 cases per 100,000 person-days. The German-speaking part had a very low incidence rate of 7.1 cases per 100,000 person-days. Detailed numbers are shown in Table 1 and are compared with the numbers in the general population. Incidence rates were between 3 and 9 times higher in dialysis patients than in the general population.

**Table 1:** Incidence rates per 100,000 person-days stratified according to geographical region in the first wave.

**Region**	**Dialysis patients with COVID-19**	**Incidence rate in dialysis patients**	**Population in CH with COVID-19**	**Incidence rate in the general population**
Italian	20	66.9	3'284	9.5
French	58	61.1	14'770	6.8
German	18	7.1	12'975	2.2
Total	96	25.5	31'029	3.7

#### Characteristics

Sixty-four positively tested dialysis patients in the first wave were male, which corresponds to a percentage of 66.7%. This number is very similar to the proportion of male dialysis patients in the general dialysis population with 65.2%. Mean age was 69.5 years, with 72.1 and 68.1 years in female and male patients, respectively (*p* = 0.250). Caucasian ethnicity was represented with 95.4%, whereas 39.6% of positive dialysis patients were diabetics and 11.5% suffered from chronic obstructive pulmonary disease (COPD). The overwhelming majority of patients (94.3%) where dialyzed in a center or a hospital, while only one home hemodialysis and four peritoneal dialysis patients were infected with SARS-CoV-2.

#### Deaths

Thirty (12 female, 18 male) out of 96 SARS-CoV-2 positive dialysis patients died during the first wave (31.3%). In 25 of these, Covid-19 was given as the cause of death, which corresponds to a mortality rate of 21.9% in men and 34.4% in women (overall 26.0%). Mortality was highest in patients suffering from hypertension/renovascular disease as primary renal disease (PRD, 41.4%), followed by diabetes (28%) and other (17.6%), and lowest in those with glomerulonephritis (0%). However, patients with glomerulonephritis were also significantly younger than those with hypertension or diabetes. All 25 patients died within one to 15 days after diagnosis of COVID-19, with a mean survival time of 7.3 ± 4.1 days. Of the remaining five patients reported dead with but not from Covid-19, causes of death were cerebrovascular incident (1), patient refusal to continue dialysis (2), withdrawal of treatment for medical reason (1) and neoplasia (1).

### Second Wave (01.10.2020–31.01.2021)

#### Number of Patients and Distribution

**Figure 4:**
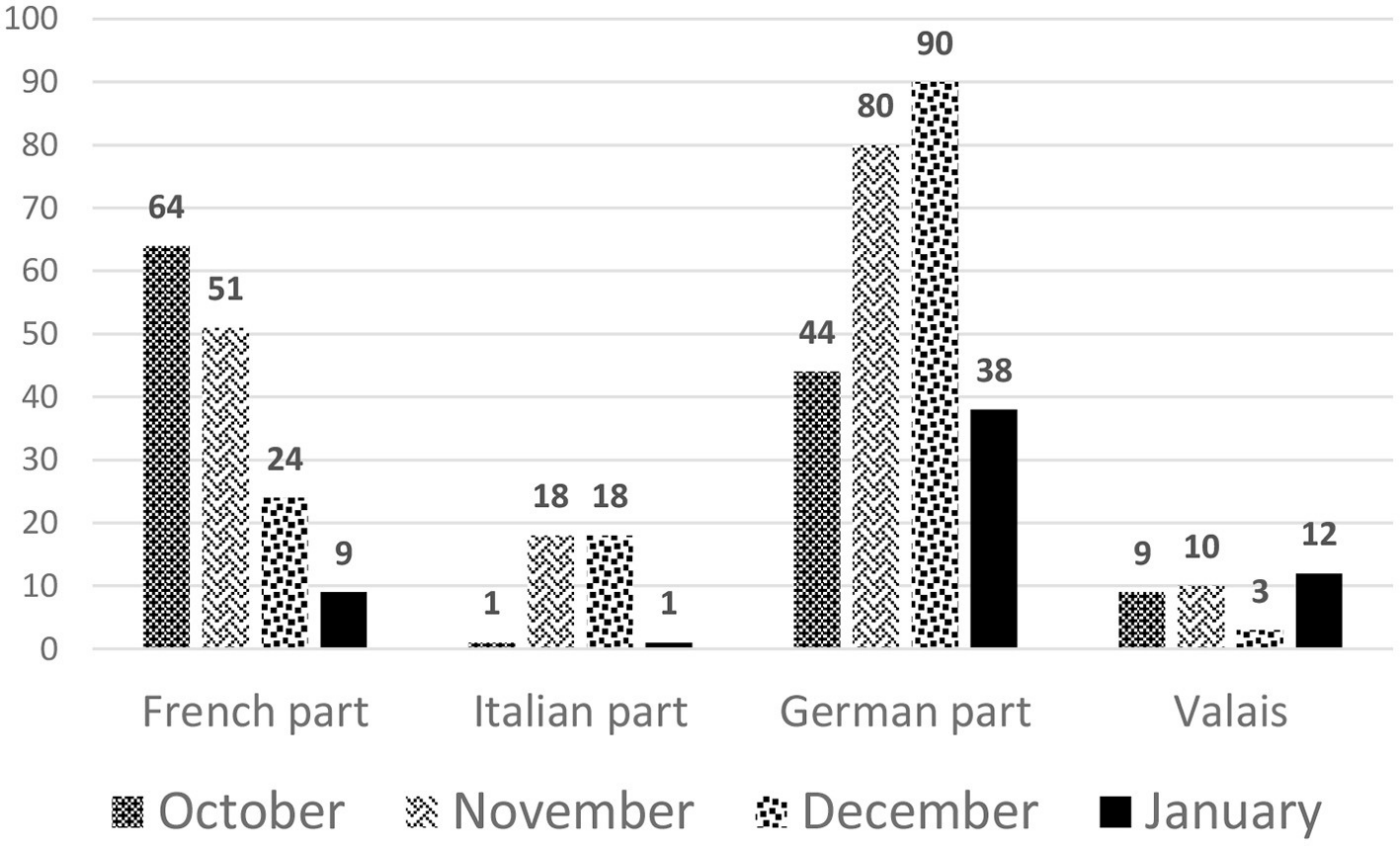
Number of SARS-CoV-2 positive dialysis patients stratified by month and region in the second wave.

**Figure 5:**
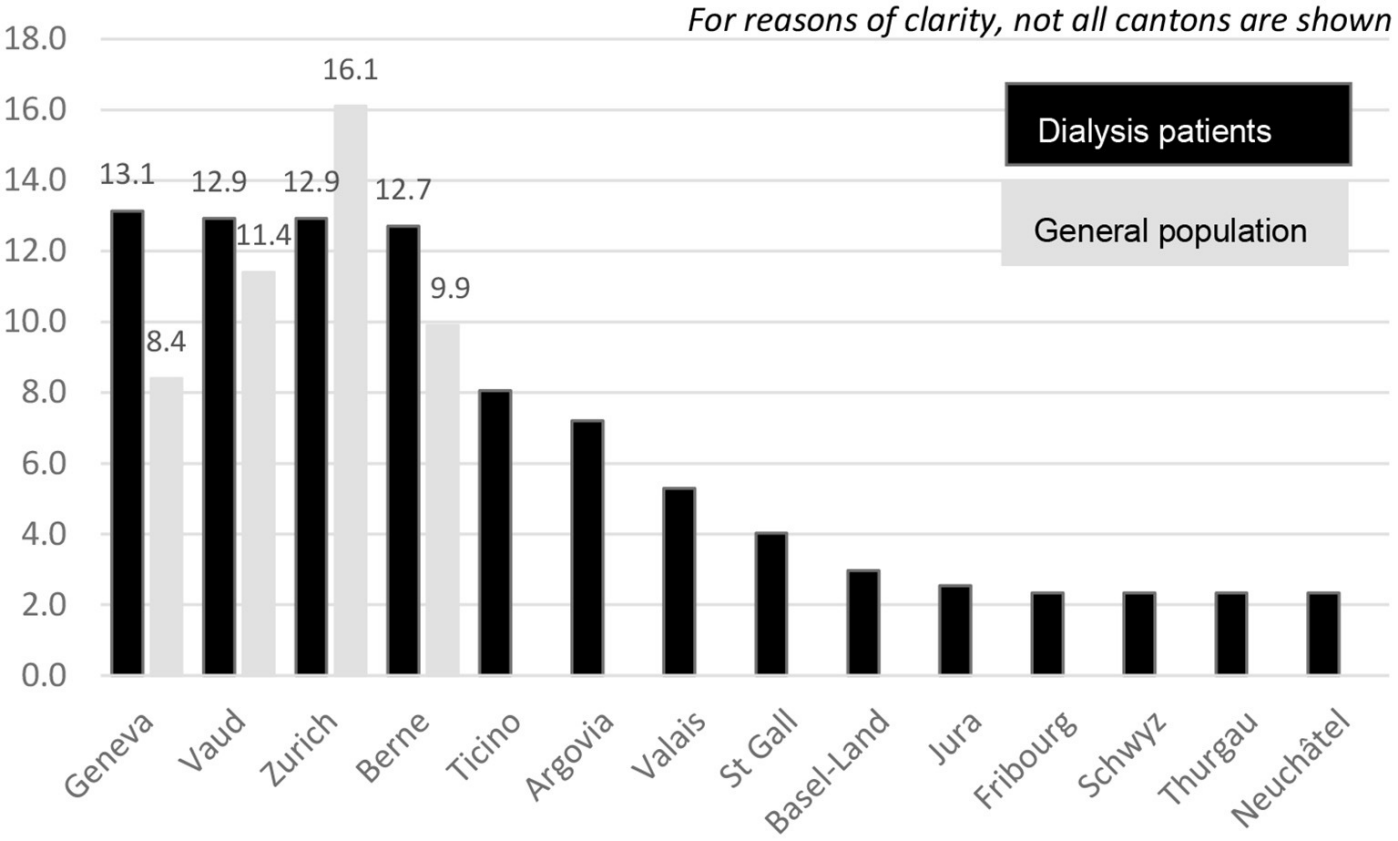
Percentage of positive SARS-CoV-2 patients in each canton during the second wave in dialysis patients (black bars) and the general population (grey bars).

Dialysis patients were hit harder during the second compared to the first wave, as was the rest of the population in Switzerland. From October 1, 2020, to the end of January 2021, 472 dialysis patients tested positive for SARS-CoV-2, as shown in Figure 4, stratified by month and region. In contrast to the first wave, the second wave affected all cantons in which a dialysis center is located (Figure 5). Incidence of Covid-19 was higher in German-speaking Switzerland compared to the first wave. Geneva was affected most during the second wave, and accounted for 13.1% of positively tested SARS-CoV-2 patients, followed by Vaud and Zurich with 12.9% each, and Berne with 12.7%, together making up for half of the SARS-CoV-2 infected dialysis patients in Switzerland (Figure 5).

During the second wave, the French speaking regions showed the highest incidence rate with 154.5 dialysis cases per 100,000 person-days. In Ticino, the incidence rate was 50 cases lower with 101.3 per 100,000 person-days compared to the French part, while the lowest incidence rate was observed in the German speaking part with 79.1 dialysis patients per 100,000 person-days.

Detailed numbers are shown in Table 2 and are compared with the numbers in the general population. During the second wave, incidence rates were 2–3 times higher in dialysis patients compared to the general population.

**Table 2:** Incidence rates per 100,000 person-days stratified according to geographical region in the second wave.

**Region**	**Dialysis patients with COVID-19**	**Incidence rate in dialysis patients**	**Population in CH with COVID-19**	**Incidence rate in the general population**
Italian	38	101.3	23,110	53.5
French	184	154.5	160,341	58.5
German	250	79.1	287,892	38.6
Total	472	99.8	471'343	44.3

#### Characteristics

The percentage of male dialysis patients tested positive in the second wave was 65.7%. Mean age was 71.2 years, with 70.5 and 71.6 years in female and male patients, respectively (*p* = 0.422). Caucasian ethnicity was represented with 90.6%, whereas 30.4% of positive dialysis patients were diabetics and 12.7% suffered from COPD. The vast majority of patients (93.4%) were dialyzed in a center or a hospital, while 1.7% of positively tested patients where on home hemodialysis and 4.9% on peritoneal dialysis.

#### Deaths

Ninety-three (26 female, 67 male) out of 472 dialysis patients died in the second wave. In 75 of these 93 deaths Covid-19 was given as the cause of death, which corresponds to a mortality rate of 17.4% in men and 13.0% in women (overall 15.9%), and was highest in patients suffering from hypertension/renovascular disease as PRD (19.2%), followed by other (16.7%), and, once more, lowest in those with glomerulonephritis (11.7%). These 75 patients died within one to 49 days, with a mean survival time of 14.0 ± 42.2 days. Of the remaining 18 patients reported dead with but not from Covid-19 infection, causes of death were cardiovascular (7), infection (2), patient refusal to continue dialysis (2), withdrawal of treatment for medical or other reason (4), neoplasia (1), gastro-intestinal hemorrhage (1) and unknown (1).

### Cumulative Results From the First and Second Wave (24.02.2020–31.01.2021)

From February 24, 2020 until January 31, 2021, 573 dialysis patients were tested positive for SARS-CoV-2 and reported to the registry.

The highest incidence rate was determined for the French-speaking part for both dialysis patients and the general population with 74.1 and 24.4 cases, respectively, per 100,000 person-days, followed by the Italian-speaking region with an incidence rate of 55.4 cases per 100,000 person-days. Detailed numbers are shown in Table 3 and compared with the numbers in the general population. Incidence rates were between 2 and 3 times higher in dialysis patients than in the general population.

**Table 3:** Incidence rates per 100,000 person-days stratified according to geographical region in both waves.

**Region**	**Dialysis patients with COVID-19**	**Incidence rate in dialysis patients**	**Population in CH with COVID-19**	**Incidence rate in general population**
Italian	58	55.4	26'736	22.2
French	246	74.1	186'282	24.4
German	271	30.7	312'490	15.0
Total	575	43.6	525'508	17.7

**Figure 6:**
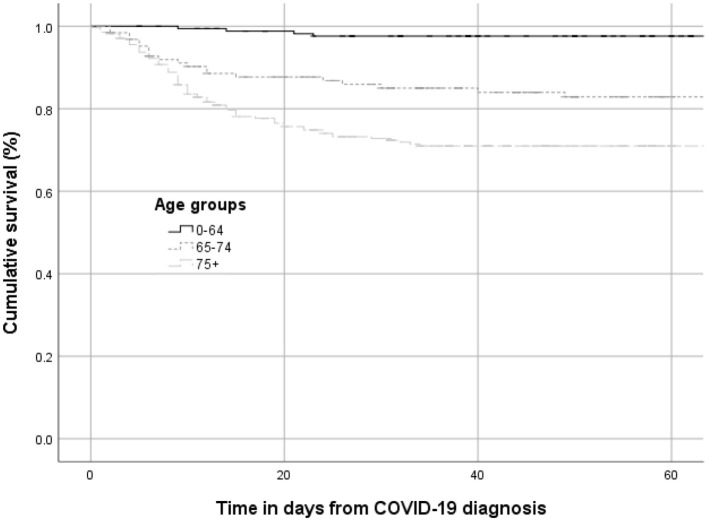
Cumulative survival (%) stratified by age groups (first and second wave).

Combined 123 (38 female, 85 male) out of 573 dialysis patients died during the two waves. In 100 of these, Covid-19 was given as the cause of death, which corresponds to a mortality rate of 18.2% in men and 16.1% in women (overall 17.5%) (Table 4). Cumulative survival according to the different age groups is presented in Figure 6. All 100 patients died within one to 49 days, with a mean survival time of 12.4 ± 9.3 days.

**Table 4:** Probability of death due to COVID-19 and risk factors in COVID-19-positive dialysis patients (both waves).

		**Probability of death within 28 days (%)**	**Hazard ratio (95% CI)**	
	***N***	**Crude**	**Crude**	Adjusted^*^
**Age at COVID-19 diagnosis**				
0–64 years	175	2.3	1	1
65–74 years	127	15.7	7.8 (2.7–22.7)	7.8 (2.7–22.7)
75+ years	271	28.0	14.6 (5.3–39.8)	14.5 (5.3–39.7)
**Sex**				
Male	374	18.2	1	1
Female	199	16.1	0.9 (0.6–1.3)	0.9 (0.6–1.3)
**Primary renal disease**				
Diabetes	121	15.7	1	1
Hypertension	180	22.8	1.5 (0.9–2.6)	1.0 (0.6–1.7)
Other	195	16.4	1.0 (0.6–1.8)	1.0 (0.6–1.9)
Glomerulonephritis	68	10.3	0.6 (0.3–1.5)	0.8 (0.4–2.0)
**Charlson Score**				
Low (2+3)	160	14.4	1	1
Mid-low (4)	104	18.3	1.3 (0.7–2.5)	1.1 (0.6–2.1)
Mid-high (5+6)	117	19.7	1.4 (0.8–2.6)	1.1 (0.6–2.0)
High (7+)	74	27.0	2.0 (1.1–3.6)	1.4 (0.8–2.6)
**Previous transplantation**				
No	415	19.3	1	1
Yes	40	0.0	0	0

* *The model for age was adjusted for sex; the model for sex/Charlson Score/ previous transplantation was adjusted for age; the model for PRD was adjusted for age and sex*.

Table 5 compares positively tested dialysis patients who died during both waves due to COVID-19 to dialysis patients who survived their COVID-19 infection. Patients dying of COVID-19 are older (80.1 vs. 68.2 years; *p* = 0.000), have more comorbidities (3.1 vs. 2.5; *p* = 0.006) and a higher Charlson Comorbidity Index (5.0 to 4.3; *p* = 0.010), are more frequently substituted with iron (88.2 vs. 75.6%; *p* = 0.012) and have no previous transplantation (0.0 vs. 10.8%, *p* = 0.002). Conversely, the percentage of diabetes and hypertension is very similar in both groups.

**Table 5:** Characteristics (given as mean±SD or percentage) according to survival status in both waves.

	**Non-Survivors,**	**Survivors,**	***p*** **-value**
	***n*** **= 100**	***n*** **= 450**	
Age, years	80.1 ± 8.1	68.2 ± 14.6	0.000
Male gender, %	68.0	64.2	0.474
Body mass index, kg/m^2^	27.5 ± 5.8	27.4 ± 5.7	0.783
Dialysis vintage, years	4.1 ± 3.3	5.4 ± 7.1	0.004
Previous transplantation, %	0.0	10.8	0.002
Residual CrCl^◦^, ml/min	3.3 ± 4.3	3.4 ± 4.2	0.863
Hemoglobin, g/dL	11.2 ± 1.4	11.1 ± 1.4	0.547
Iron substitution, %	88.2	75.6	0.012
Comorbidities, *n*	3.1 ± 1.9	2.5 ± 1.9	0.006
Charlson Comorbidity Index	5.0 ± 2.3	4.3 ± 2.0	0.010
Diabetes, %	34.0	32.0	0.699
Hypertension^*^, %	83.5	86.4	0.495
COPD, %	12.9	12.2	0.848
Neoplasia, %	20.0	14.7	0.184

* *Diagnosis of hypertension;*

◦ *Creatinine Clearance*.

Logistic regression analysis identified age as the only significant risk factor for mortality in dialysis patients. Notably, we did not observe male sex, diabetes or COPD to be major risk factors associated with mortality in dialysis patients in Switzerland.

Table 6 gives an overview of characteristics for COVID-19 positive and negative patients, respectively. Positive patients were older (70.8 vs. 68.7 years; *p* = 0.001), had a higher body mass index (BMI; 27.3 vs. 25.8; *p* = 0.000), were longer on dialysis (5.2 vs. 4.0 years; *p* = 0.000), are less likely to be dialyzed at home (6.6 vs. 10.9%; *p* = 0.004), suffer less from diabetes or COPD (31.8 vs. 37.6%; *p* = 0.007, and 12.3 to 15.9%; *p* = 0.043, respectively) and are more frequently substituted with iron (78.0 vs. 72.7%; *p* = 0.008) than COVID-19-free dialysis patients.

**Table 6:** Characteristics (given as mean±SD or percentage) of dialysis patients with and without COVID-19.

	**With COVID-19**	**Without COVID-19**	***p*** **-value**
	**(*n* = 573)**	**(*n* = 4,249)**	
Age, years	70.8 ± 14.3	68.7 ± 14.8	0.001
Male gender, %	65.3	65.4	0.941
Body mass index, kg/m^2^	27.3 ± 5.7	25.8 ± 5.7	0.000
Dialysis vintage, years	5.2 ± 6.7	4.0 ± 4.0	0.000
Dialysis duration per week (h)	11.4 ± 1.3	11.5 ± 1.5	0.301
1–2 dialysis sessions/week or PD, %	9.1	12.4	0.042
Home dialysis^*^, %	6.6	10.9	0.004
Kt/V	1.56 ± 0.36	1.59 ± 0.42	0.065
Residual function, ml/min	3.4 ± 4.2	3.9 ± 4.8	0.110
Hemoglobin, g/dL	11.1 ± 1.4	11.1 ± 1.4	0.996
Ferritin, ng/mL	496 ± 336	512 ± 444	0.463
Calcium, mmol/L	2.2 ± 0.2	2.2 ± 0.2	0.840
Phosphate, mmol/L	1.6 ± 0.5	1.6 ± 0.5	0.128
PTH, ng/L	350 ± 308	369 ± 324	0.228
Comorbidities, n	2.6 ± 1.9	2.6 ± 2.0	0.678
Charlson Comorbidity Index	4.4 ± 2.1	4.5 ± 2.2	0.718
Hypertension, %	85.3	83.0	0.210
Diabetes mellitus, %	31.8	37.6	0.007
COPD, %	12.3	15.9	0.043
Iron substitution, %	78.0	72.2	0.008
Erythropoietin substitution, %	81.8	79.5	0.249

* *Home hemodialysis and peritoneal dialysis*.

### Third (01.02.2021-30.06.2021), Fourth (01.07.2021-14.10.2021) and Fifth Wave (15.10.2021-28.02.2022)

During the third wave, 61 SARS-CoV-2 positive dialysis patients were recorded in the dialysis registry. As during the second wave, the incidence rate was highest in the French part of Switzerland with 15.8 dialysis cases per 100,000 person-days. The overall incidence rate was 10.6 dialysis cases per 100,000 person-days. In contrast to the previous waves, the incidence among the general population is now higher than in dialysis patients, with 14.1 cases per 100,000 person-days. Only six deaths due to COVID-19 were reported, which corresponds to a mortality rate of 9.8%.

During the fourth wave, 43 dialysis patients developed COVID-19. Incidence rate was the same as in the third wave with 10.6 dialysis cases per 100,000 person-days. However, German-speaking regions showed the highest incidence rates with 14.4 dialysis cases per 100,000 person-days for the first time during the pandemic. Incidence was 1.6 times higher in the general population compared to dialysis patients with 17 cases per 100,000 person-days. Only one death was reported to the registry in the fourth wave, corresponding to a mortality of 2.3%.

In the fifth wave, 443 dialysis patients were infected with SARS-CoV-2. This corresponds to an incidence rate of 105.7 cases per 100,000 person-days. The incidence also increased enormously in the general population, reaching 215.5 cases per 10,000 person-days and being twice as high as in the dialysis population. As in the second and third wave, incidence was highest in French-speaking Switzerland with 92.3 cases per 100,000 person-days. 13 dialysis patients died due to COVID-19 in the fifth wave, which corresponds to a mortality rate of 2.9%.

As numbers were low, no survival analyses were performed for the third, fourth and fifth wave.

## Discussion

This is the first report to provide a complete overview of the COVID-19 pandemic among dialysis patients across Switzerland. Our analyses reveal four interesting findings: first, the incidence of SARS CoV-2 infections showed a high heterogeneity among regions and a much higher incidence rate in maintenance dialysis patients compared to the general population in the first and second wave; second, mortality in dialysis patients with COVID-19 was increased compared to the general population and other European countries; third, only age was a significant independent risk factor for mortality in COVID-19 dialysis patients, but not male sex as shown in other studies (11). Fourth, incidence and mortality were much lower during the third, fourth and fifth wave, reflecting the impact of early vaccination of this vulnerable population.

Several reasons may explain the higher incidence rates during the first and second wave in dialysis patients. First, maintenance dialysis patients were systematically screened. Most dialysis centers measured body temperature in their patients before starting the dialysis session. Thus, patients with minor symptoms were identified immediately. In the general population, however, testing was more restrictive and limited, especially in the beginning of the pandemic with a lower detection among mild cases. Testing strategies in the general population changed during the second wave, with increased testing even of persons with mild symptoms. However, asymptomatic people were still hard to identify and not tested systematically. Second, dialysis patients being dependent on regular treatment sessions often using public transportation exposed themselves to the risk of community transmitted infection. Third, contact with health care workers is also a potential source of transmission, what is supported by the fact that patients on 1–2 dialysis sessions per week or on PD were tested less often positive for SARS-CoV-2 than patients on thrice-weekly dialysis schedules (Table 6). A systematic review and meta-analysis from the first wave from Nopsopon et al. (12) supports our findings with end-stage renal disease (ESRD) patients getting tested positive much more often than the general population.

Dialysis patients and the general population in the Italian-speaking part of Switzerland were most affected by the pandemic during the first wave compared to the rest of Switzerland (Table 1). This may be explained by the many Italian migrant workers, crossing the border from northern Italy, where the virus was active much earlier, and thus was brought to the southern Switzerland first to be carried from there across Switzerland. In the second wave, the incidence among dialysis patients was highest in French-speaking Switzerland, followed by Italian-speaking Switzerland, and the German part of Switzerland (Table 2). There is no evident explanation for these differences in incidence rates among dialysis patients from the various regions in Switzerland. Strict screening policies were introduced shortly after the start of the outbreak in all regions: body temperature was measured and patients were systematically asked about possible symptoms before each dialysis session and before entering the dialysis unit. Anyone with symptoms was isolated and underwent PCR-testing. The structure of dialysis units is rather similar throughout Switzerland, with a large capacity to isolate potentially infected persons. Wearing of surgical masks was and still is mandatory for dialysis staff and patients. During the first wave, the Swiss Civilian Service even assured transport toward dialysis centers for some patients who had lost their usual means of transportation (e.g., Red Cross voluntary drivers who were forced to interrupt usual work) in order to avoid public transport. The most likely reason, however, is the higher incidence rate in the general population of Ticino and French-speaking Switzerland, increasing the risk for dialysis patients from these regions to be exposed to the Coronavirus.

In the Swiss dialysis population, 26% of patients with COVID-19 died in the first wave, whereas during the second wave the percentage was only 15.9%. This may be due to potential underdiagnosis of individuals with only mild infection in the first wave. An alternative explanation is the higher awareness and earlier and more appropriate therapeutic intervention, which is supported by the longer mean survival time of almost 1 week in the second wave. Overall, 100 COVID-19-deaths were registered from 573 positively tested dialysis patients (17.5%) in the first two waves. In the general population, mortality of COVID-19 patients was much lower, with 5.5% in the first, 1.5% in the second wave, and 1.7% overall (2). The higher mortality rate among dialysis patients can be explained by several factors: first, their frailty; second, their much higher average age compared to the general population; and, third, more restricted admission of dialysis patients to intensive care unit (ICU) treatment because of severe disease (13). This possibility is supported by the policy of some hospitals such as the university hospital of Geneva (HUG), where many patients who died from COVID-19 would not have been admitted to an ICU because of local triage criteria (6). Our findings, again, are supported by the above mentioned review and meta-analysis (12), which showed that ESRD patients have a 3.6 times higher risk of dying than the general population in the first wave.

Unlike in the publication from Jager et al. (11), only higher age was associated with mortality in our study. We could not confirm male sex, diabetes or COPD being risk factors for death in dialysis patients with COVID-19 as found in the general population (14). This is in contrast to dialysis patients without COVID-19, where male individuals show a slightly increased mortality compared to women (15). None of the forty previously transplanted patients on dialysis died. This finding may be a coincidence, but could also be linked to the relative protection offered by low-dose corticosteroid therapy against adverse outcome due to COVID-19. Such an association has been recently suggested for transplanted patients, but whether low dose prednisone therapy offers the same protection in dialysis patients is unknown. Clearly, the number of previously transplanted patients was too low analyze this result further, and more research in larger cohorts is necessary to draw definite conclusions.

Interestingly, dialysis patients with Covid-19 in Switzerland (together with the Netherlands) had the highest probability of death (11) compared to other European countries in the first wave. The strict measures taken in France, Spain and Austria may have contributed to the fact that fewer dialysis patients in these countries have died. However, analyzing both waves, dialysis patients in Switzerland had a lower COVID-19 attributable mortality with 17.5% compared to dialysis patients from other European countries with 20.0% (11). This better survival rate in dialysis patients in Switzerland has already been shown in several annual reports from the ERA-EDTA (European Renal Association – European Dialysis and Transplant Association) independently of the COVID-19 pandemic (16).

In Switzerland, vaccination of particularly vulnerable individuals started on January 4, 2021, including dialysis patients. The second vaccine in Switzerland (from Moderna) was approved on January 12, 2021 (17). The decline in positive cases among dialysis patients during the third, fourth and fifth wave probably reflects the positive effect of early vaccination in this population. The finding that only 20 deaths were reported since February 1, 2021, strongly suggests that vaccination protects the dialysis population thoroughly from severe courses and death.

Our study has several limitations: First, the number of patients from the first wave is relatively small, which makes it difficult to compare the first with the second wave. Second, using registry data as a source, we had no access to additional information on more specific patient and treatment characteristics that could be important for the outcome of COVID-19 patients on dialysis, and data were not directly captured by the authors, but by the treating nephrologists. Third, matching the reported dialysis patients that were tested positive with the information entered in the registry, we realized that about 50 patients were missing, and, therefore, not included in our analyzes.

## Summary and Conclusion

The COVID-19 pandemic challenged the dialysis centers in Switzerland with a much higher incidence rate of infections and a very high mortality rate compared to the general population in the first and second wave. The application of measures such as wearing masks, systematic measurement of the temperature before entering the dialysis center and isolation of suspected cases probably helped to decrease the difference in incidence between the dialysis population and the general population, but the measures were insufficient, and only vaccination lead to a steep decrease in numbers. It is of utmost importance to further explore how this vulnerable group can be better protected from future outbreaks or further pandemics. In addition, more studies are needed to investigate whether and why positive tested previously transplanted dialysis patients seem to be better protected than dialysis patients who have had no transplant. In order to limit severe infections in vaccinated dialysis patients, repetitive booster vaccinations may be advisable.

## Data Availability Statement

The raw data supporting the conclusions of this article will be made available by the authors, without undue reservation.

## Ethics Statement

The studies involving human participants were reviewed and approved by Kantonale Ethikkomission Zürich, Switzerland. Written informed consent from the participants' legal guardian/next of kin was not required to participate in this study in accordance with the national legislation and the institutional requirements.

## Author Contributions

RG drafted the manuscript, reviewed literature, and responsible for data collection and participated in data interpretation. MP critically revised the manuscript and participated in data interpretation. PA supervised the study, critically revised the manuscript, and participated in data interpretation. All authors contributed to the article and approved the submitted version.

## Conflict of Interest

The authors declare that the research was conducted in the absence of any commercial or financial relationships that could be construed as a potential conflict of interest.

## Publisher's Note

All claims expressed in this article are solely those of the authors and do not necessarily represent those of their affiliated organizations, or those of the publisher, the editors and the reviewers. Any product that may be evaluated in this article, or claim that may be made by its manufacturer, is not guaranteed or endorsed by the publisher.

## Abbreviations

SARS-CoV-2: Severe Acute Respiratory Syndrome Coronavirus 2
COVID-19: Coronavirus disease 2019
WHO: World Health Organization
SRRQAP: Swiss Renal Registry Quality Assessment Program
HD: Hemodialysis
PD: Peritoneal dialysis
COPD: Chronic obstructive pulmonary disease
PRD: Primary renal disease
CrCl: Creatinine Clearance
BMI: Body mass index
ICU: Intensive care unit
ERA-EDTA: European Renal Association — European Dialysis and Transplant Association
ESRD: End-stage renal disease.
